# Forward Light Scattering of First to Third Generation Vitreous Body Replacement Hydrogels after Surgical Application Compared to Conventional Silicone Oils and Vitreous Body

**DOI:** 10.3390/gels9100837

**Published:** 2023-10-21

**Authors:** Maximilian Hammer, Jonathan Herth, Marcel Muuss, Sonja Schickhardt, Alexander Scheuerle, Ramin Khoramnia, Grzegorz Łabuz, Philipp Uhl, Gerd Uwe Auffarth

**Affiliations:** 1 David J Apple Laboratory for Vision Research, 69120 Heidelberg, Germany; maximilianhammer@icloud.com (M.H.);; 2 Department of Ophthalmology, University Clinic Heidelberg, 69120 Heidelberg, Germany; 3 Institute for Pharmacy and Molecular Biotechnology, 69120 Heidelberg, Germany

**Keywords:** hydrogel, vitreous body, stray light, forward light scattering, click chemistry, vitreoretinal surgery, retinal surgery

## Abstract

To treat certain vitreoretinal diseases, the vitreous body, a hydrogel composed of mostly collagen and hyaluronic acid, must be removed. After vitrectomy surgery, the vitreous cavity is filled with an endotamponade. Previously, pre-clinical hydrogel-based vitreous body substitutes either made from uncrosslinked monomers (1st generation), preformed crosslinked polymers (2nd generation), or in situ gelating polymers (3rd generation) have been developed. Forward light scattering is a measure of Stray light induced by optical media, when increased, causing visual disturbance and glare. During pinhole surgery, the hydrogels are injected into the vitreous cavity through a small 23G-cannula. The aim of this study was to assess if and to what extent forward light scattering is induced by vitreous body replacement hydrogels and if Stray light differs between different generations of vitreous body hydrogel replacements due to the different gelation mechanisms and fragmentation during injection. A modified C-Quant setup was used to objectively determine forward light scattering. In this study, we found that the 1st and 3rd generation vitreous body replacements show very low stray light levels even after injection (2.8 +/− 0.4 deg^2^/sr and 0.2 +/− 0.2 deg^2^/sr, respectively) as gel fragmentation and generation of interfaces is circumvented. The 2nd generation preformed hydrogels showed a permanent increase in stray light after injection that will most likely lead to symptoms such as glare when used in patients (11.9 +/− 0.9 deg^2^/sr). Stray light of the 2nd generation hydrogels was 3- and 2-fold increased compared to juvenile and aged vitreous bodies, respectively. In conclusion, this significant downside in the forward light scattering of the 2nd generation hydrogels should be kept in mind when developing vitreous body replacement strategies, as any source of stray light should be minimized in patients with retinal comorbidities.

## 1. Introduction

The vitreous body is a transparent gel located in the vitreous cavity between the lens and retina [1]. Its hyaluronic acid and collagen form a complex three-dimensional structure to support the retina [2]. Beyond stability, the vitreous body aids ocular tissue metabolism, storing glucose, lactic acid, and antioxidants to nourish the surrounding tissue [3].

In cases where its integrity is compromised, such as in vitreoretinal pathologies, surgical intervention becomes necessary. Vitrectomy is used to treat a variety of ophthalmological diseases, such as floaters [4], retinal detachments [5], proliferative vitreoretinopathy [6], or endophthalmitis [7]. During vitrectomy, mostly three small gauge trocars (23 or 25 Gauge) are used to perform surgery minimally invasive. During surgery, the vitreous body is carefully removed from the eye by using a cutter that cuts the vitreous body into small parts that can be sucked out of the eye. The vitreous body has to be carefully removed from its adhesion to the retina. Once all vitreous is shaved off the retina, the vitreous cavity has to be filled with an endotamponade to stabilize the retina [8]. Clinically used replacement strategies are silicone oils [9,10], different kinds of gases, a balanced salt solution, or air [11]. The oil’s surgical [12,13], physicochemical [14], and pharmacological properties [15] have been extensively studied and improved on in the past: High-molecular weight polydimethylsiloxane (PDMS) was added to improve the tendency to emulsify, semi fluorinated alkanes were added to PDMS to modify the oil’s density to treat inferior pathologies and improve the pharmacokinetics of drugs [16,17]. However, many downsides of the oil use, such as the emulsification of the silicone oil in the vitreous cavity, origin from its lipophilic character, which is why major efforts have been undertaken to develop hydrogel vitreous body substitutes [18,19,20,21,22,23], which have been directly compared to silicone oils in animal studies.

**Figure 1:**
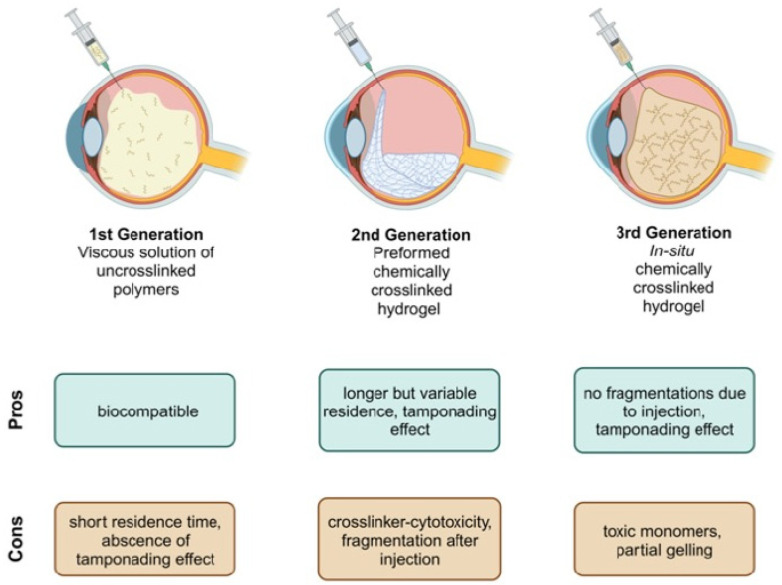
**Overview of vitreous body replacement hydrogels.** The different vitreous body replacement strategies have distinct advantages and disadvantages. There is no previous information on forward light scattering for any vitreous body replacement hydrogel.

The hydrogel vitreous body replacement strategies have evolved from viscous solutions of uncrosslinked polymers as the first generation, through the second generation of preformed chemically crosslinked hydrogels, to the third generation of in situ chemically crosslinked hydrogels (Figure 1) [18].

The first generation describes viscous solutions of uncrosslinked polymers, which are routinely used as ophthalmological viscoelastic devices (OVDs) during cataract surgery [24]. However, they show too short residence duration, an unsuitable swelling pressure, and a lack of tamponading force to replace the vitreous body [25,26]. Thus, while they were initially developed as a vitreous body replacement, their use is limited to the intraoperative time frame. Even a small amount of OVD not removed from the eye at the end of surgery can lead to intraocular pressure spikes due to blockage of the trabecular meshwork and due to swelling [27,28]. Currently, a large variety of OVDs is in use. Most of them are composed of hyaluronic acid as a polymer. Various OVDs with different molecular weights are available, commonly referred to as cohesive or dispersive OVDs based on their intraoperative properties, as introduced by Arshinoff [29] and Poyer and colleagues [30]. As the monomers are not crosslinked, intermolecular entanglement plays an important role when designing a new OVD [30]. Currently, available products contain 1% to 3% hyaluronic acid [24]. Additionally, new alternatives are made from hydroxypropylmethylcellulose [31].

The 2nd established generation is performed by chemically crosslinked hydrogels [20,21,32,33]. These gels have a long residence duration and provide tamponading force. However, it is unclear how the optical properties of these hydrogels are altered due to fragmentation during the small gauge injection process. Most often, crosslinking solutions are added to gelate the polymers in vitro [19,21,34]. This leaves the hydrogels vulnerable to fragmentation during the surgical implantation of the gels through a 23G trocar system. A trend towards even smaller-gauge vitrectomy to up to 27G surgery [35,36,37] may even aggravate fragmentation. Next to fragmentation, safety measures due to toxicity and inflammation have to be in place to guarantee biocompatibility, which can be challenging [34,38,39,40]. A variety of monomers have been used in the past, such as alginate [33], hyaluronic acid [34], polyethylene gylcole [41], and tetra-polyethylene glycole [19].

**Figure 2:**
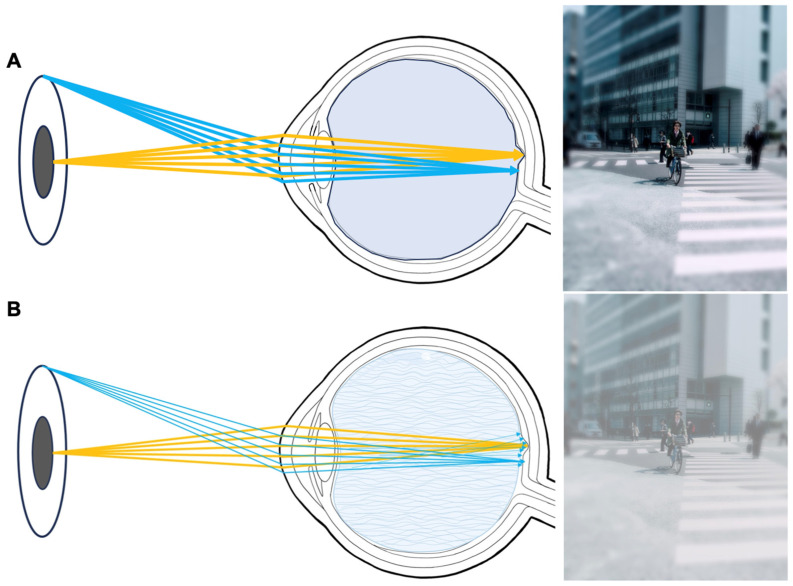
Compared to in situ gelling vitreous body replacements (**A**), e.g., the 3rd generation vitreous body replacements), preformed gels will undergo fragmentation, causing the development of interfaces within the vitreous cavity that can induce stray light (**B**). Increased stray light can lead to symptoms such as hazy vision and glare, as presented on the right. Prior to this study, forward light scattering had never been evaluated for vitreous body replacement strategies.

Third-generation vitreous bodies circumvent these concerns by chemically crosslinking the hydrogels in situ. In this case, many positive properties of hydrogels are combined, but the monomers must be extensively tested for toxicity since they gel in situ [19,42]. In the past, multiple reaction types, including click chemistry [19], aldehyde condensation [43], and Schiff base reactions [42], have been examined. Next to transparency, turbidity, and the refractive index, forward light scattering is an important parameter to measure in potential vitreous body substitutes to assess the predicted optical disturbance and glare that patients may experience [44,45]. A scattering of the light beam towards the retina, which can be caused by finely dispersed impurities, interfaces, or opacification of the gels, causes it to no longer focus on the retina [46]. Figure 2 showcases an illustration of the situation a patient might experience if the ocular stray light is increased. This important optical parameter has been previously studied in ophthalmology only in regard to intraocular lenses [44]. It was never evaluated for vitreous body replacement strategies.

In this study, we analyzed forward light scattering of previously published vitreous body replacement strategies of all three generations before and after injection through a 23G-cannula as clinically applied. Stray light was compared to currently used silicone oils and porcine as well as human vitreous bodies to set results into the current clinical perspective. For the 1st generation replacement, we chose a clinically used viscoelastic device consisting of uncrosslinked hyaluronic acid. For the 2nd generation, we chose a crosslinked hydrogel based on alginate because of its wide availability and to test a different monomer. For the 3rd generation, we chose a previously published Tetra-PEG hydrogel because it is one of the few in-situ gelating hydrogels that remained optically clear in a rabbit eye for a year without showing signs of toxicity.

## 2. Results and Discussion

### 2.1. Results

After gelation, all vitreous body replacement strategies underwent an organoleptic assessment. The specimen presented as transparent, colorless, odor-free hydrogels. No localized opacifications or calcifications were apparent upon visual examination.

**Chemical and viscoelastic properties of the tested hydrogels**

pH measurements were conducted for all hydrogels. The following pH was measured for the 1st to 3rd generation vitreous body replacement hydrogels, respectively: 6.96, 6.8, and 4.99. For the 2nd and 3rd generation hydrogels, additionally, the monomeric solutions were measured, reaching a pH of 5.41 and 5.08 (for both high thiol and high maleimide pregels).

**Figure 3:**
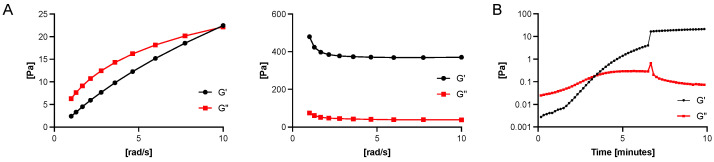
Viscoelastic properties of the 1st to 3rd generation vitreous body replacements strategies. (**A**) depicts a frequency sweep of the 1st (**left**) and 2nd (**right**) vitreous body replacement hydrogel. (**B**) depicts the sol-gel transition occurring within minutes after combining the pre-gels. After only around four minutes, the gel point is reached. The reaction allows the safe and quick in-situ crosslinking inside the vitreous cavity.

Figure 3 shows the viscoelastic properties and viscosity of the tested substances. For the 3rd generation of vitreous body replacement hydrogel, the sol-gel transition was recorded to examine the speed of gelation after injection.

**All vitreous body replacements show low levels of stray light before fragmentation.**

**Figure 4:**
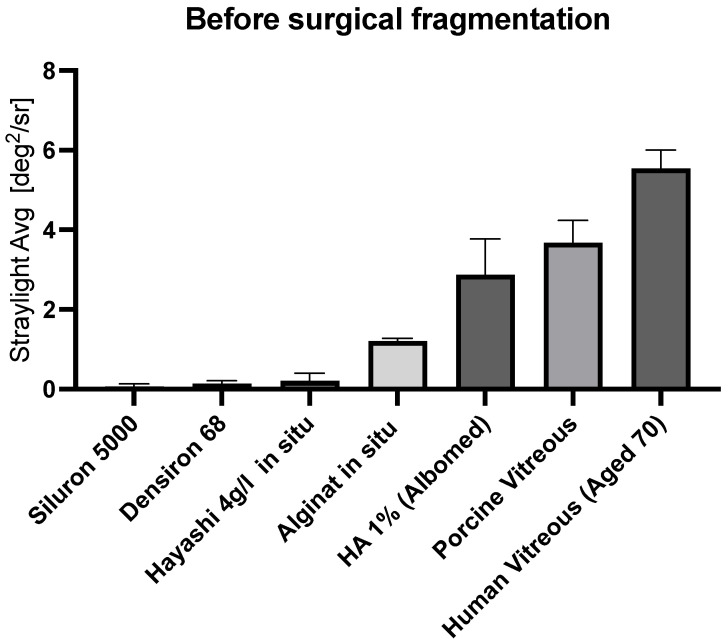
Baseline stray light of different vitreous body replacement strategies and juvenile (porcine) and aged (human) vitreous bodies. Prior to the injection process, all vitreous body replacements showed a lower stray light than the natural vitreous body.

All vitreous bodies were synthesized and stray light-evaluated. All replacement strategies showed very low amounts of stray light. Silicone oils had the lowest stray light, followed by the 3rd, 2nd, and 1st generation vitreous body replacement hydrogels. Prior to fragmentation by injection, all replacement strategies induced less stray light than the porcine and aged human vitreous body (Figure 4).

**Fragmentation induced by small-gauge injection greatly induces permanent stray light in the 2nd generation hydrogel but not in the 1st and 3rd generation hydrogels.**

**Figure 5:**
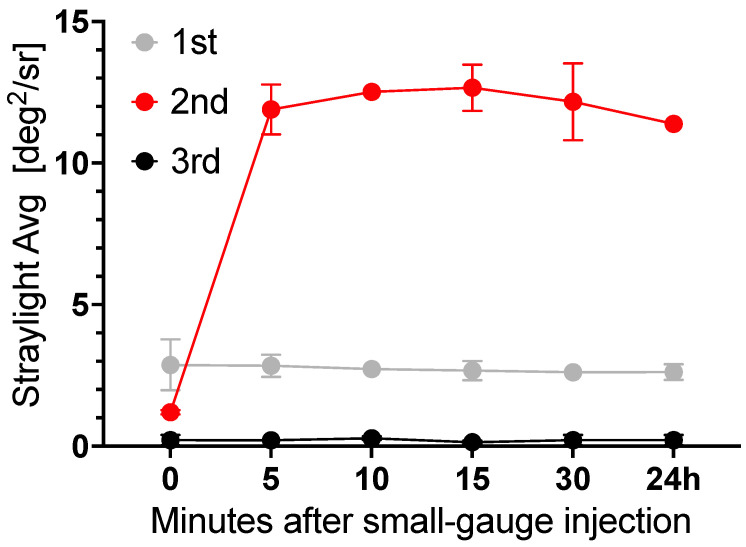
**Impact of small-gauge injection on forward light scattering of the 1st, 2nd, and 3rd generation vitreous body replacement hydrogels.** The 1st and 3rd generation vitreous body replacement strategies showed no increase in stray light. For the 3rd generation replacement strategies, gelation took place within 10 min after the injection process. As such, gel fragmentation occurred. The 2nd generation hydrogel showed a major increase in stray light.

After injection through a 23G-polyimide cannula, stray light of the vitreous body replacement strategies was evaluated. For the 3rd generation of hydrogel, both components were mixed and injected through the cannula, as applied in porcine in vivo studies to overcome gel fragmentation. Thus, gelation again occurred in the UV-cuvette [19]. Fragmentation introduced visible interfaces in second-generation hydrogels immediately after injection. Stray light did not recover over a 24-hour period. The 1st and 3rd generation hydrogels showed no changes in stray light before and after injection (Figure 5).

**Clinically used and novel vitreous body replacement strategies compared to the vitreous body.**

**Figure 6:**
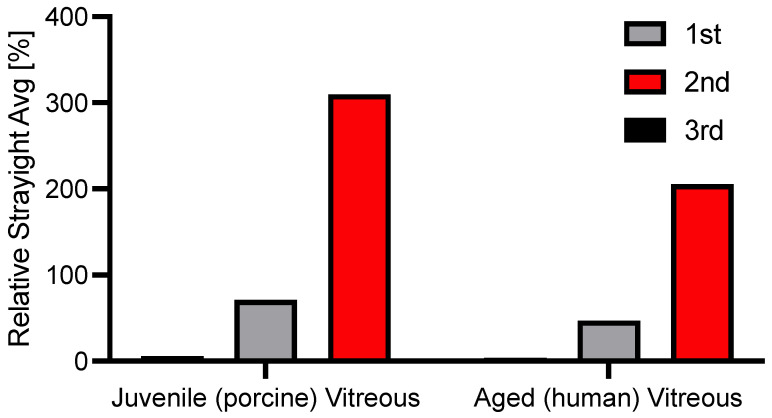
Comparison of stray light induced by the hydrogels to juvenile (porcine) and aged (70-year-old human) vitreous bodies after the injection process.

**Figure 7:**
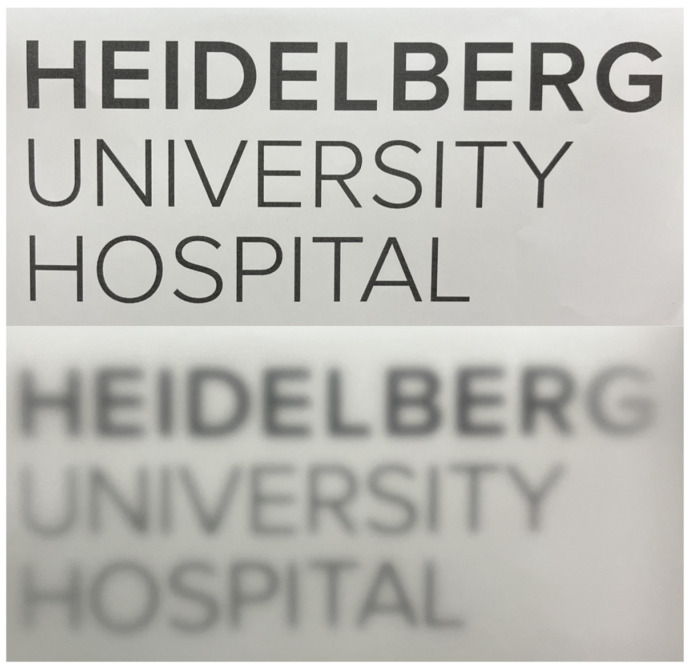
**Illustration of visual disturbance comparing the 3rd generation (top) and the 2nd generation (bottom) vitreous body replacement hydrogels.** The photographs of the logo were taken at a distance of 1 m with 1 × 1 cm cuvette filled with the respective hydrogel in front of a camera with constant shutter speed and aperture. The fragmentation likely causes changes in the refraction as well, further aggravating possible symptoms.

The natural vitreous body is a near-optically clear gel. This is, up to our knowledge, the first study to ever evaluate forward light scattering in explanted vitreous bodies. To better compare stray light values of novel vitreous body substitutes, they were set into perspective with juvenile (porcine) and aged (human) vitreous bodies. The 2nd generation vitreous body replacement gel showed three times greater stray light than the juvenile vitreous body after fragmentation induced by injection through a 23G-cannula. The difference is illustrated in Figure 6. Figure 7 depicts what a patient might experience.

### 2.2. Discussion

#### 2.2.1. Summary

In this study, we compared the stray light induced by the 1st to 3rd generation vitreous body replacements to porcine and human vitreous bodies as well as current long-term endotamponades, namely heavier-than-water and lighter-than-water silicone oil. Results were compared after gelation and after gel fragmentation through the injection of a 23G-cannula commonly used during vitreoretinal surgery. This is the first study to date to assess forward light scattering of the vitreous body and gel replacement strategies.

#### 2.2.2. Forward Light Scattering

Forward light scattering has been used to assess the optical impact of implants in ophthalmology, especially for intraocular lenses made from different hydrophilic and hydrophobic acrylic materials [44,47]. However, the technology has not been applied to hydrogels and materials of posterior segment surgery. The highest stray light values in our studies were seen for a 2nd generation vitreous body replacement after fragmentation with a mean of 11.4 deg^2^/sr, roughly three times the amount of stray light induced by a juvenile, porcine vitreous body (see Figure 6 and Figure 7). To set these results into perspective, this amount of stray light is induced by the average crystalline lens of a 70-year-old patient (mean: 11.2 deg^2^/sr), as previously reported [48]. Increased forward light scattering can lead to symptoms such as glare and reduced contrast sensitivity, compromising daily activities. Given that patients receiving vitreous body replacements most likely have significant retinal comorbidities, additional sources of stray light should be minimized.

#### 2.2.3. Reasons for the Increase in Stray Light after Gel Fragmentation

Only the 2nd generation’s vitreous body replacement hydrogel showed an increased stray light after injection through a 23G retinal surgery cannula. As it is the only tested hydrogel that was crosslinked prior to the injection, gel fragmentation is very likely the cause of this increase. The injection introduces new gel-aqueous humor interfaces that cause light to scatter at each interface. HA-mono-mers, as the 1st generation vitreous body replacement hydrogels, quickly form the same viscous, homogenous gel after injection. Thus, no additional interfaces where light is scattered occur. Similarly, by circumventing fragmentation via in situ gelation as applied in the 3rd generation of vitreous body replacement hydrogels, an increase in forward light scattering is prevented. Similarly, heavier- and lighter-than-water silicone oils form one silicone oil bubble in the vitreous cavity after injection. The unique application of hydrogels as a vitreous body demands the highest optical clarity, low light scattering, and a refractive index close to the natural vitreous body. While it is known that additional interfaces, as present in, e.g., emulsions, majorly influence stray light, prior to this study, it was unclear to what extent the injection process of vitreous body substitutes degrades their optical performance. This study shows a possible weakness of the 2nd generation, and thus preformed/ crosslinked, hydrogels in replacing the vitreous body.

In this study, we also conducted the first reported in vitro measurements of the natural porcine and, thus, juvenile and 70-year-old human vitreous bodies. The vitreous body is composed of different collagens, hyaluronic acid, and other glycosaminoglycans. During the aging process, the association between collagen and hyaluronan is altered, causing liquefication and fibrous degeneration [2]. Previously, only one in vivo study in human subjects conducted forward light scattering measurements focusing on the vitreous body, specifically on myodesopsia [49]. Castilla-Marti et al. showed that in patients with monocular floaters, collagen aggregates in the aged vitreous body and a sign of vitreous aging, higher stray light values can be observed compared to the healthy partner eye. In line, the human-aged vitreous showed higher stray light than the juvenile porcine vitreous body of pigs aged 8–9 months. Previously, only the viscoelastic but not the optical properties of the vitreous body were evaluated in detail in vitro. Filas et al. showed that vitreous body aging could be simulated by enzymatic degradation using G′ and G″ as well as their ratio as the outcome [50]. Similarly, Schulz et al. examined human vitreous bodies of different ages and were able to show a clear decline in viscoelastic properties of the vitreous body with increasing age [51]. Further studies should evaluate the optical change of enzymatic degradation of the vitreous body.

This study shows minor limitations. First, we only tested one hydrogel per generation. It is possible that other hydrogels exhibit a different level of stray light after fragmentation. Additionally, all experiments were conducted in vitro. Therefore, the severity of possible symptoms can only be extrapolated.

In conclusion, the 3rd generation of vitreous body replacement hydrogels shows favorable in vitro stray light by circumventing fragmentation during the surgical injection process. Preformed, crosslinked hydrogels may induce stray light, and thus glare and haziness, when used as a vitreous endotamponade. Forward light scattering in explanted vitreous bodies might be an approach to further study myodesopsia and develop therapies to revert optical changes of the vitreous body.

## 3. Conclusions

In conclusion, 3rd generation vitreous body replacement hydrogels show favorably in vitro stray light by circumventing fragmentation during the surgical injection process. Preformed, crosslinked hydrogels may induce stray light, and thus glare and haziness, when used as a vitreous endotamponade. Forward light scattering in explanted vitreous bodies might be an approach to further study myodesopsia and develop therapies to revert optical changes of the vitreous body.

## 4. Materials and Methods

The chemical composition of all examined hydrogels and silicone oils is presented in Table 1.

### 4.1. Materials

Silicone oils were provided by Fluoron GmbH, Ulm, Germany. Heavier- and lighter-than-water silicone oils were examined in this study, namely Siluron 5000 and Densiron 68 (Table 1). The PDMS in Densiron 68 is equivalent to Siluron 5000, which is why we used Densiron 68 as a direct comparison. Pe-Ha-Luron^®^ F 1.0% was purchased from Albomed GmbH, Schwarzenbruck, Germany. Alginate solution (0.5%) was purchased from Alginatec GmbH, Riedenheim, Germany. 4ARM-SH-10K (M = 10 kg/mol) and 4ARM-MA-10K (M = 10 kg/mol) were purchased from JenKem Technology (Tianjin, China).

#### 4.1.1. Porcine and Human Vitreous Body Preparation

The vitreous bodies of 8 eyes from 4 pigs aged 8 to 9 months (Schradi Frischfleisch GmbH, Mannheim, Germany) were carefully removed. The animals were not solely killed for this study. During the removal, special care was taken to maintain the original three-dimensional structure of the porcine vitreous body [51,52]. An incision at the equator was made first, and then the vitreous body was carefully moved out of the vitreous cavity using blunt instruments. Six human vitreous bodies were explanted in a similar fashion from the donor’s eyes from the adjunct cornea bank of the University Eye Hospital Heidelberg. This study was approved by the local ethics committee (S-134/2018). Post-mortem written consent was obtained from relatives. For this study, six vitreous bodies aged 70 years of age were used to compare the vitreous stray light of juvenile (porcine) to aged (human) vitreous bodies.

#### 4.1.2. First Generation Hyaluronic Acid Gel

Pe-Ha-Luron 1%, an ophthalmic viscoelastic device made from 1% hyaluronic acid [53], was used as the 1st generation vitreous body replacement. The molecular weight of the monomers is between 1.2–2.0 million Dalton. The viscosity of the solution after steam sterilization is around 20,000 mPas with an osmolality of 270–400 (mOsm/kg). Next to sodium hyaluronate, sodium chloride, disodium hydrogen phosphate 2 H_2_O, and Sodium dihydrogen phosphate 2 H_2_0 are included in the product.

#### 4.1.3. Second Generation Alginate Gel

Five milliliters of alginate solution (0.5%, Alginatec) were transferred into a dialysis membrane (8 kDa, Ø 11.5 mm; Spectra/Por^®^ 7 Dialysis Membrane, Repligen, Boston, MA, USA) and crosslinked for 4 h at room temperature by placing it in an aqueous 11.6 mM calcium sulfate dihydrate solution, chemical details were previously described by Russo et al. [54]. The dialysis membrane was washed with the balanced salt solution, as previously described by Schulz et al. [9].

#### 4.1.4. Third Generation Tetra-PEG Gel

As presented by Hayashi et al. [19], a pre-gelating process was conducted, creating Oligo-Tetra-PEGs to reduce the in situ gelling time. Respectively, with concentrations of 12.6 g/L and 7.4 g/L, both substances were dissolved in citrate-phosphate buffer (pH 5.0, di-Sodium hydrogen phosphate dihydrate and Citric acid monohydrate). Equal volumes of the higher concentration (12.6 g/L) of 4ARM-SH-10K and the lower concentration (7.4 g/L) of 4ARM-MA-10K, as well as vice versa, were mixed and left to react for at least 12 h. To measure the forward light scattering, equal amounts of both mixed solutions were combined in a UV cuvette [10]. Crosslinking occurs spontaneously through the thiol-maleimide-click reaction without a catalyst in situ [55].

#### 4.1.5. Measurement of forward Light-Scattering

To quantify stray light, we used a technique initially introduced by van der Berg and colleagues [46] for clinical assessment of ocular stray light. This methodology was subsequently adapted by Łabuz et al. [45] for the in vitro evaluation of intraocular lenses and has been embraced by our research team for light scattering analysis. A modified commercially available diagnostic device, the C-Quant (Oculus, Wetzlar, Germany), was mounted with a custom-designed optical setup. This configuration enabled consistent and unbiased assessment of stray light. Figure 2 illustrates the fundamental concept of in vivo stray light measurement. The C-Quant device utilizes a psychophysical compensation comparison approach to measure ocular stray light. A specialized attachment for the C-Quant instrument ensured that only the sample was exposed to the stray light source, while the observer eye’s contribution was eliminated by a field-stop mechanism. Consequently, the observer can evaluate the light diffused by the object without any influence from the observer’s eye-generated stray light. Stray light measurements were conducted by two operators (M.H, J.H.), both of whom were unaware of the characteristics of the samples to ensure a blind procedure.

#### 4.1.6. Study Setup

Stray light of all vitreous body replacement hydrogels was evaluated when synthesized in the measurement cuvette and after injection into a measurement cuvette through a 23G-cannula. After injection, stray light was evaluated immediately and after 5, 10, 15, and 30 min, as well as after one day. Gels were sealed and stored in a humid environment overnight to prevent evaporation.

#### 4.1.7. Measurement of Viscosity and Viscoelastic Properties

The gels were placed on the plate of the rheometer (Anton Paar MCR, 302e, Anton Paar, Graz, Austria). The second plate was then lowered to a distance of 1 mm. Viscosity measurements were conducted at 25 °C. Frequency sweeps were performed from 1 to 10 rad/s. Depending on the substance to be tested, roughened plates were used, as previously described, for the measurement of highly hydrated substances, such as the bovine vitreous body, to reduce wall slip [50].

#### 4.1.8. Sol-Gel-Transition of the 3rd Generation Vitreous Body Replacement Hydrogel

Tetra-PEG-SH and Tetra-PEG-MA were individually dissolved in citrate-phosphate buffer (pH 5.0, di-Sodium hydrogen phosphate dihydrate, and Citric acid monohydrate).) to achieve a concentration of 10 g/L for each. These solutions were left to stand for a minimum of 12 hours. Subsequently, the two solutions, Tetra-PEG-SH 10 g/L and Tetra-PEG-MA 10 g/L were combined and injected into the gap between the cone-plate setup of the rheometer (Anton Paar MCR 302e, Anton Paar, Graz, Austria). The oscillatory shear rheological properties, including the storage modulus (G′) and the loss modulus (G″), during gelation, were measured at 25 °C using the cone-plate setup for thirty minutes to determine the Sol-gel-transition.

#### 4.1.9. pH-Measurements

The pH value was measured using a pH meter (FiveEasy, Mettler Toledo). The calibration was performed with buffer solution pH 7.0 (Fluka Analytical, Honeywell Research Chemicals, Morris Plains, NJ, USA) and buffer solution pH 4.0 (Fluka Analytical, Honeywell Research Chemicals, Morris Plains, NJ, USA).

## Figures and Tables

**Table 1:** Components and mechanisms of gelation of all tested endotamponade solutions.

Name	Monomer/Compounds	Mechanism of Gelation	Reason for Use
G1: Hyaluronic Acid 1%	High molecular weight Hyaluronic Acid (1.200–2.000 kDa)	Only monomers, no gelation	Clinically used in anterior segment surgery
G2: Crosslinked Alginate	Alginate (1000 kDa) 11.6 mM calcium sulfate dihydrate solution	Crosslinked by complexing alginate via Ca^2+^	One of the first G2 strategies, well-characterized
G3: Oligo-Tetra-PEG	Tetra-PEG functionalized with thiol and maleimide functional groups	Crosslinked via click-chemistry of different oligomers	Remained clear for one year in rabbit eyes without toxicity
Siluron 5000	100% Polydimethylsiloxane	Only monomers, no gelation	Currently clinically used
Densiron 68	30.5% F6H8, 69.5% Polydimethylsiloxane	Only monomers, no gelation	Currently clinically used

Abbreviations: G1–G3, 1st to 3rd generation vitreous body replacement.

## Data Availability

Data are available upon reasonable request from the corresponding author GUA.
